# A Short Overview of the Effects of Kinesio Taping for Postural Spine Curvature Disorders

**DOI:** 10.3390/jfmk3040059

**Published:** 2018-11-27

**Authors:** Francesca Borzì, Marta Anna Szychlinska, Michelino Di Rosa, Giuseppe Musumeci

**Affiliations:** Department of Biomedical and Biotechnological Sciences, Human Anatomy and Histology Section, School of Medicine, University of Catania, 95123 Catania, Italy

**Keywords:** Kinesio Taping, spine deviation, postural control, proprioception disorder, rehabilitation, prevention

## Abstract

Spine curvature disorders are very common in the population. Several therapeutic methods have been implemented over time. Kinesio Taping (KT) is a solution that is utilized for several purposes. This narrative review aims to discuss KT methodology as a valid solution for spinal curvature disorders, especially for structured and non-structured spine deviations. The matter is poorly discussed in the current literature. Nevertheless, KT seems to indirectly influence posture and spine curvature disorders through peripheral and central nervous system stimulation, but further investigations are needed to demonstrate these unknown effects clearly. The present review provides a valuable contribution to the existing literature and may represent a starting point and a useful guide for further studies in this field of research.

## 1. Introduction

**Figure 1:**
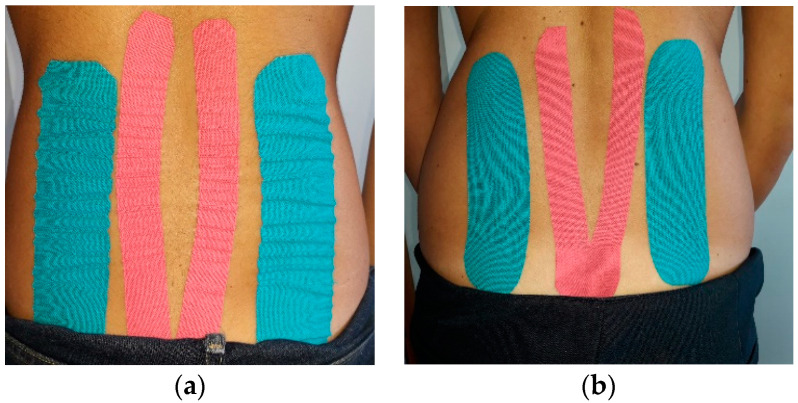
Examples of KT on spine: (**a**) taping with no tension for decompressive application; (**b**) taping with tension for postural application.

Studies in the field of conservative treatment of spine curvature disorders increased in frequency after a long period of progressive decline lasting from the 1980s to the early 2000s. This is important since orthopedists are increasingly engaged in surgical training and performance, and less of their attention goes to conservative treatment [1]. Spine curvature disorders are a very common occurrence among the population from childhood to old age [2,3]. There are three main types of spine curvature disorders: (1) lordosis, also called swayback, where the spine curves significantly in the anterior direction in the lower back; (2) kyphosis, which is characterized by an abnormally rounded upper back (more than 50 degrees of curvature); and (3) scoliosis, characterized by a lateral curve to the spine. The curve is often S-shaped or C-shaped. Structural alterations to the spine may lead to functional disturbances that, in turn, cause pain, inflammation, muscle weakness, nervous system stimuli disorders, and injuries [4]. Spine deviations are classified as “paramorphisms” and “dysmorphisms”. The former (from Greek “παρα µορφη” meaning “close to the shape”) are skeletally non-structured spine curvatures, while the latter (from Greek “δυσμορφία” meaning “deformity”) are skeletally structured spine curvatures. One of the most common spine deviations is scoliosis. According to the Society on Scoliosis Orthopaedic and Rehabilitation Treatment (SOSORT), a share of 2–3% of the population suffer from adolescent idiopathic scoliosis [5]. It may be a predisposing determinant for degenerative conditions of the spine in adulthood [6,7]. The onset could be determined by several factors including muscle weakness due to low physical activity, inappropriate posture as well as improper transportation of school equipment [8]. Broadly, it consists of a combination of incorrect postural habits and muscle hypo/hyper-activity [4]. Posture is a widely studied matter, as is its interaction with Kinesio Taping (KT) methods. KT methods are based on the use of acrylic adhesive tape characterized by different colors, widths, skin-similar thicknesses, and tensile forces. It is waterproof and air permeable. One of the most important KT functions is lifting the skinfold, which, in turn, promotes blood circulation and lymphatic flow [9,10,11,12]. The physiological alteration leads to more efficient drainage as well as a reduction in swelling and reabsorption of hematomas [9,10,11,12]. Initially, athletes’ injuries were treated by non-elastic tape to maintain and restore balance without joint mobility limitations [13]. Later, KT was designed to provide free range of motion (ROM) and stability for joints through the tensile forces created in conjunction with the wave-like grain and the adhesive surface [13]. Over time, several types of tape have been created to satisfy several purposes, such as prevention, rehabilitation and performance improvement, each with a different application technique [11,13]. According to Kase [10], for muscle inhibition or muscle relaxation, the tape is applied from the muscle insertion to its origin, with the tension being weaker than 15–25% of the original tension, while for muscle strength, tape is applied from the muscle origin to the insertion, with tension stronger than 25–50% (Figure 1) [10]. However, other schools do not share the same application technique [10,14]. KT may not be capable of instantaneously modifying strength production in healthy players, but could have an important positive result on muscle fatigue resistance during frequent concentric muscle actions [14,15,16,17]. Additionally, the potential beneficial effects of KT on muscle endurance should not be ignored either [15]. Several studies have shown good efficacy and positive effects of KT on pain [16,17]. In detail, in both the first research group [16] found that KT users had an amelioration in pain compared to the control group. In the latter [17], where groups were additionally sorted according to the tension/no tension application, a similar result was found. KT has an effect on other pain-related disorders, such as osteoarthritis (OA). In fact, authors recently showed the positive effect of KT on joint ROM, pain, swelling, and muscle force in subjects affected by knee OA [18,19]. Conversely, a detailed study carried out by Wagek et al. [20] proved that there was no amelioration in terms of pain, swelling, or muscle force in subjects affected by knee OA. Less controversial are studies about the positive effect of KT on pain secondary to post-training muscle damage [21,22]. KT application was demonstrated to have a beneficial effect on delayed onset of muscle soreness after eccentric muscle contractions involving both lower and upper limbs [22]. Quicker recovery post-training [21,22,23] as well as the restoration of myofascial pain [24] were also reported. The neuromuscular system, proprioception, and posture are, in turn, affected by KT, as reported in a survey conducted in infants suffering from cerebral palsy [25]. Movement control was a key finding of a study conducted in patients suffering from axial dystonia, and it turned out that the symptoms were ameliorated by KT application [26]. Similar results were achieved in patients with hemiplegia secondary to stroke [27,28] as well as in patients with Parkinson’s disease, who showed ameliorations in axial postural disorders, including body posture alterations, body unbalance, and walking disability, in an ad-hoc rehabilitative protocol based on proprioceptive stimulation provided by KT [29]. Several studies focused on spine disorders that, as mentioned above have been determined to involve several factors, including muscle weakness, muscle unbalance [4,8], and also pain [30] and proprioception alteration [31]. Studies on KT application have been conducted over the years; however, most of them have focused on the lower back only [2,26,30], and just a few have concerned the possible effects on other spine regions and spine deviations in general [30,31,32,33,34,35,36,37]. The aim of this narrative review is to investigate what the literature reports with regard to the application of KT in spine curvature disorders and to determine whether this method may achieve such a substantial and beneficial effect as to be considered as an integrative and non-invasive method to be implemented in addition and/or substitution to traditional rehabilitation programs.

## 2. Kinesio Taping and Spine

A review of the current literature shed light on particular matters regarding KT and the spine. Spine questions are represented by lower back disorders, which are the most studied conditions, probably because of their high incidence, and because they are considered an important public health problem in many countries [2,3,4,5,38]. These disorders are associated with considerable direct and indirect costs [38]. Studies on the lower back and KT have mostly investigated muscle strength, pain, mobility limitations, and disability [39,40,41,42,43]. Research by Alvarez and coauthors [39] proved the positive effect of KT on the lower back muscle fatigue by demonstrating an enhancement of extensor lumbar muscle resistance, an important factor for low back pain management. This result was also confirmed by Hagen and colleagues [40] and Castro-Sanchez and colleagues [41], who affirmed that one week of KT application positively influences pain, disability, and muscle endurance, even if some of the outcomes are not maintained over time. Furthermore, Preece and colleagues [37], explained the immediate improvement of trunk forward flexion in patients with a history of non-specific lower back pain through the KT effect. In contrast, in recent research, it was shown that there was no amelioration within 24 hours in chronic low-back pain subjects after KT applications [42]. This finding was supported also by Parreira and coauthors [43], who found no significant effects on pain intensity, or disability from using the KT method, regardless of the application technique. Postural control of the lower back is positively affected by KT application as well [30,33,36,37,44]. A study conducted on sacroiliac joint dysfunction showed that the KT method balances pelvic inclination and sacral horizontal angle, thereby influencing postural control [30]. Tests on postural control and KT effect were also performed on pregnant women. Pregnancy intensifies lumbar lordosis and abdominal and gluteal muscle weakness resulting in body posture changes and low back pain [31]. Only two studies have been conducted on this population, each with a different KT application technique [32,33]. On one hand, the results indicated immediate efficiency (follow up and intervention were done after few days), but on the other they did not predict long lasting effects [32,33]. KT has been tested as an integrative method in physiotherapy for spine disorder treatment, but there are conflicting opinions. The authors of [34] demonstrated that the therapeutic effect of spinal manipulation is not enhanced by KT application in chronic non-specific low back pain management in young and adult athletes. However, this study’s findings are in contrast with findings reporting that KT is more effective than manipulation treatment or sham taping on hip muscles activation for improving physical performance and pain management in patients suffering from patellofemoral syndrome [45]. Kamali et al. [34] utilized KT to test a multimodal approach to the treatment of low back pain, a method already suggested by Gonzales-Iglesias and colleagues [35] in 2009, who hypothesized the association of KT with already proved efficient interventions, such as exercise therapy in patients suffering from the acute effects of whiplash. Moreover, the KT intervention was compared with cervical thrust manipulation in patients with neck pain, and it turned out that both methods have positive, even clinically relevant, effects on pain relief, but KT has less effect on ROM and disability [46]. Other studies were conducted on cervical spine disorders. Copurgensli et al. [36] discovered that the use of KT in association with Mulligans’s mobilization has more positive effects on patients affected by cervical spondylolisthesis compared to the conventional rehabilitation. There are no studies concerning the comparison between KT and bracing for the treatment of spine deformities. It has been shown that the progression of structural spine deviation should slow down or stop through physical therapy and bracing [47,48,49]. The latter is designed to treat the three-dimensional nature of the deviation according to biomechanical principles in order to satisfy not only aesthetic but also functional needs [47,48,49]. The effects of spinal braces on postural control in different sensory conditions in adolescent idiopathic scoliosis was investigated [50]. Despite the limited motion caused by the rigidity of the spinal brace, it was proven that, over time, subjects improved their postural stability in terms of increased proprioception, equilibrium performance, and rhythmic movement ability [50], suggesting that there was a body perception reprogramming. However, the real efficacy of bracing is a matter of discussion, especially regarding physical and physiological functions and psychological outcomes [51,52,53]. Thus, it is not possible to exclude in advance that KT may exert a similar effect to an increase in proprioception without side effects.

## 3. Possible Explanation for the Effects of KT

**Figure 2:**
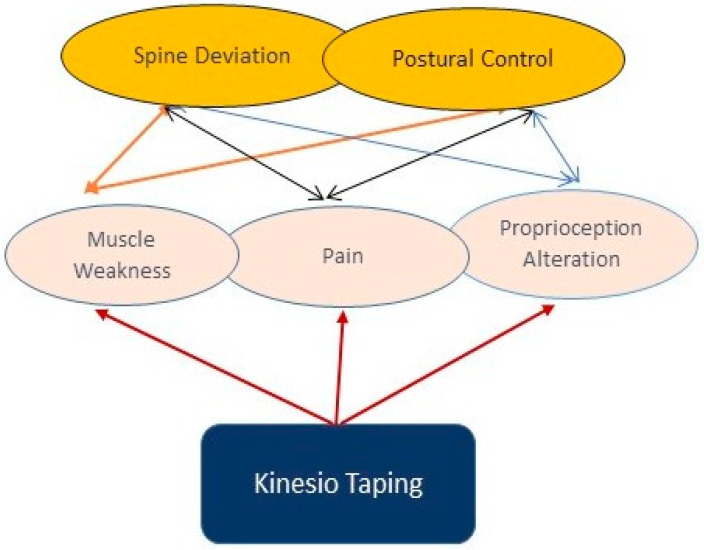
Schematic graph illustrating the indirect influence of Kinesio Taping (KT) on spine disorders and postural control [4,8,27,29,30,31].

There are several possible explanations regarding the efficacy of the KT method. Firstly, it could be explained by the placebo effect [54,55] as well as by sensory stimulation secondary to skin traction [56]. The traction exerted by the tape on the epidermis enhances the pressure on the dermis mechanoreceptors leading to a decrease in nociceptive stimuli [13,56]. In more detail, fibrous connective tissue has numerous mechanoreceptors that are occasionally activated to transmit sensitive and short-lived impulses. If these activations are prolonged, they quickly become oversensitive and even painful. Although individuals can withstand the initial painful symptoms, the condition may be persistent and have a significant recurrence rate [57]. Since KT leads to a mechanical stimulus of pressure for skin deformation, it results in a decrease of nociceptor activation. A study conducted by Cimino and colleagues [58] examined the skin deformation caused by KT application on the low back, and it demonstrated the enhancement of skin stretch at the end of the tape and a retraction along the edges, coupled by a reduction in skin thickness at the end and an increase along its edges. However, no results were found regarding the hypodermal thickness, and the magnitude of the effects were also postural-dependent. Thus, it is possible to hypothesize that (1) deactivation of sensitive short-lived impulses and thus pain reduction occurs because of the decrease in skin stretch that generates proper mechanic stimulation; and (2) there is a decrease in swelling as the fluid is kept away from the affected area because of the changes in skin thickness [58]. Exemplifying studies were conducted by Lins et al. [56] and Lemos et al. [59], who reported on sensory stimulation and connective tissue modifications leading to improvements in flexibility; these results have become even more significant over time. The question about the real cause of the muscle function enhancement is controversial. Authors studied the immediate effect of forearm Kinesio Taping on maximal grip strength and force sense in healthy collegiate athletes and found that the subjects that used KT regularly had enhanced grip strength compared to those who did not use it [60]. They stated that strength improved through the feeling of an enhanced perception of muscle force. However, the results had no test validation. Bischoff and colleagues [61] proved several improvements in different regions, including proprioception, as a result of KT application in subjects affected by anterior cruciate ligament rupture. According to the findings reported above, it is possible to hypothesize that other indirect mechanisms might explain the results (Figure 2). Tactile stimulation could be one of these mechanisms, but it is limited by the fact that subcutaneous fat tissue alters KT functioning [58]. Finally, activation of the nervous system by KT application on the skin has been investigated. It consists of peripheral nerve stimulation enhancement, leading to motor cortex excitability [62] coupled with the reduction of motor neuron threshold, resulting in easier recruitment of the motor units [63]. In a recent study, the authors demonstrated the placebo effect of the facilitation of Kinesio Tape on muscle activity and muscle strength [18]. Facilitators of KT promote maximal grip strength only among regular KT users, but its effect is trivial. Interestingly, this effect is not related to any electrophysiological changes in the muscle on which KT is applied, which may indicate an indirect working mechanism leading to the increased grip strength. However, the results should be considered to be the outcome of several determinants, such as the methods and materials implemented as well as the intervention administration and outcome detection.

## 4. Discussion

The effects of the Kinesio Taping method on spinal curvatures disorders have not been thoroughly studied yet. KT has rapidly become a recognized therapeutic modality in many musculoskeletal and neurological disorders. Its properties make it a very suitable tool for the management of scoliosis or other postural curvature disorders. KT studies have reported positive effects on pain, ROM, balance, strength, function, and proprioception [64]. Applications of KT are used for: assistance of patients to ’hold’ their corrected posture (Schroth method, Scientific Exercises Approach to Scoliosis), in both idiopathic adolescent scoliosis and adult scoliosis patients; relief of pain in (elderly) scoliosis patients with postural collapse; and assistance with pulmonary function in patients with idiopathic adolescent scoliosis or neuromuscular scoliosis [64,65]. KT is capable of reducing pain when applied with or without tension and improving disability, even after its withdrawal, when applied with tension [41,66]. Several controversial studies have shown positive or inconsistent effects of KT on spine curvature disorders, body perception, and movement control, although they are limited to sectorial spine regions or individuals with musculoskeletal injury [67]. The KT method could have positive effects on non-structural spine deviations (paramorphism) as a preventive method before these deviations become structural (dysmorphisms), even if in the current literature there is a lack of studies to confirm and strengthen this hypothesis. However, some studies have not been conducted on a sufficiently large cohort of patients to give statistically significant results [15,32,33,44,68,69,70]. Furthermore, it is necessary to also consider the non-standardized KT applications where application is done according to the therapist or practitioner’s discretion. Furthermore, even if therapies that do not include KT applications have sometimes yielded satisfactory results, it is interesting to note that some individuals do not tolerate particular physical interventions well (for example the improvement of ROM through physical therapy exercises), and KT could represent a good solution as an alternative method. The limitations of this review are the absence of a systematic approach like the PRISMA Statement or similar to provide a more balanced view on the current state of knowledge in this research arena; the small number of relevant published studies on postural curvature disorders; and the lack of information regarding the method of applying KT on the body areas. This review describes the results of recent studies on the impact of KT on spine curvature disorders. It shows that this area is not well known and clarification of how various KT applications could be used to support treatment patients with specific and unspecific spine disorders is required. The use of the tape provides an easy cost-effective feedback method for trainers, patients, and therapists to cue immediate changes in spine posture during sagittal plane movements [71].

## 5. Conclusions

In conclusion, treating the causes of spine curvature disorders and not just the symptoms, using functional and non-invasive methods such as KT, could be a more advantageous choice for patients. Thus, in light of this, KT has a great influence on body posture, suggesting that some early non-structured spine deviations could be treated by the KT application method instead of classical orthopedic corsets, of course in association with adapted kinesiotherapy. KT could also represent a possible solution that avoids the side effects of bracing, but this hypothesis needs to be confirmed by long-term studies. It is not possible to firmly state that KT is a valid solution for postural spine curvature disorders, especially for structured spine deviations, but it could be conceivable for unstructured spine deviations. Nevertheless, KT seems to indirectly influence posture and spine curvature disorders through the peripheral and central nervous system stimulation, but further investigations are still needed to clearly demonstrate this unknown effect. The present narrative review provides a valuable contribution to the existing literature and may represent a starting point and a useful guide for further studies in this field of research. KT could be a wonderful new extra tool in the management of postural spine curvature disorders at different ages as a preventive intervention. It is necessary to conduct further research to determine the effect of Kinesio Taping on spinal curvature disorder, to thus determine its possible applications in selected spinal malfunctions and diseases.

## References

[1] Raciborski F., Gasik R., Klak A. (2016). Disorders of the spine. A major health and social problem. Reumatology.

[2] Coenen P., Smith A., Paananen M., O’Sullivan P., Beales D., Straker L. (2017). Trajectories of Low Back Pain from Adolescence to Young Adulthood. Arthritis Care Res..

[3] Alshami A.M. (2015). Prevalence of spinal disorders and their relationships with age and gender. Saudi Med. J..

[4] Czaprowski D.S.Ł., Stoliński Ł., Tyrakowski M., Kozinoga M., Kotwicki T. (2018). Non-structural misalignments of body posture in the sagittal plane. Scoliosis Spinal Disord..

[5] Negrini S., Donzelli S., Aulisa A.G., Czaprowski D., Schreiber S., De Mauroy J.C., Diers H., Grivas T.B., Knott P., Kotwicki T. (2018). 2016 SOSORT Guidelines: Orthopaedic and rehabilitation treatment of idiopathic scoliosis during growt. Scoliosis.

[6] Braccialli L.M.P., Vilarta R. (2000). Aspectos a serem considerados na elaboração de programas de prevenção e orientação de problemas posturais. Rev. Paul. Educ. Fis..

[7] Detsch C., Candotti C.T. (2000). A incidência de desvios posturais em meninas de 6 a 17 anos da cidade de Novo Hamburgo. Rev. Mov..

[8] Sedrez J.A., Da Rosa M.I., Noll M., Medeiro Fda S., Candotti C.T. (2015). Risk factors associated with structural postural changes in the spinal column of children and adolescence. Rev. Paul Pediatr..

[9] Aguilar-Ferrándiz M.E., Castro-Sánchez A.M., Matarán-Peñarrocha G.A., García-Muro F., Serge T., Moreno-Lorenzo C. (2013). Effects of kinesion taping on venous symptoms, bioelectrical activity of the gastrocnemius muscle, range of ankle motion, and quality of life in postmeno pausal women with chronic venous insufficieny: A randomized controlled trial. Arch. Phys. Med. Rehabil..

[10] Kase K., Wallis J., Kase T. (2003). Clinical Therapeutic Application of the Taping Method.

[11] Williams S., Whatman C., Hume P.A., Sheerin K. (2012). Kinesio taping in treatment and preevention of sports injuries: A meta-analysis of the evidence for its effectiveness. Sports Med..

[12] Shah M., Julu P.O.O., Monro J.A., Coutinho J., Ijeh C., Puri B.K. (2018). Neuromuscular taping reduces blood pressure in systemic arterial hypertension. Med. Hypotheses.

[13] Kase K., Tatsuyuki H., Tomoki O. (1996). Development of Kinesiotape. Kinesiotaping Perfect Manual.

[14] Serrao J.C., Mezencio B., Claudino J.C., Soncin R., Miyashiro S.P.L., Sousa P.E., Borges E., Zanetti V., Phillip I., Mochizuki L. (2016). Effect of 3 different applications of Kinesio Taping Denko on electromyographic activity: Inhinibion or facilitation of the quadriceps of males during squat exercise. J. Sport Sci. Med..

[15] Hagen L., Helbert J.J., Dekanich J., Koppenhaver S. (2015). The effect of elastic therapeutic taping on back extensor muscle endurance in patients with low back pain: A randomized, controlled, crossover trial. J. Orthop. Sports Phys. Ther..

[16] Ekiz T., Aslan M.D., Özgirgin N. (2015). Effects of kinesio tape application to quadriceps muscle on isokinetic muscle stegnth, gait and functional parameters in patients with stroke. J. Rehabil. Res. Dev..

[17] Aydoğdu O., Sari Z., Yurdalan S.U., Polat M.G. (2017). Clinical outcome of kinesio taping applied in patients with knee osteoarthritis: A randomized controlled trial. J. Back Musculiskelet. Rehabil..

[18] Castrogiovanni P., Di Giunta A., Guglielmino C., Roggio F., Romeo D., Fidone F., Imbesi R., Loreto C., Castorina S., Musumeci G. (2016). The effects of exercise and kinesio tape on physical limitations in patients with knee osteoarthritis. J. Funct. Morphol. Kinesiol..

[19] Musumeci G. (2017). Sarcopenia and exercise “The State of the Art”. J. Funct. Morpholog. Kinesiolog..

[20] Wageck B., Nunes G.S., Bohlen N.B., Santos G.M., De Noronha M. (2016). Kinesio Taping does not improve the symptoms or function of older people with knee osteoarthritis: A randomised trial. J. Physiother..

[21] Boobphachart D., Manimmanakorn N., Manimmanakorn A., Thuwakum W., Hamlin M.J. (2017). Effect of elastic taping, non-elastic taping and static stretching on recovery after intensive eccentric exercise. Res. Sports Med..

[22] Kim J., Kim S., Lee J. (2016). Longer application of kinesio taping would be beneficial for exercise-induced muscle damage. J. Exerc. Rehabil..

[23] Lee Y.S., Bae S.H., Hwang J.A., Kim K.Y. (2015). The effects of kinesio taping on architecture, strength and pain of muscles in delayed onset muscle soreness of biceps brachii. J. Phys. Sci..

[24] Öztürk G., Külcü D.G., Mesci N., Şilte A.D., Aydog E. (2016). Efficacy of kinesio tape application on pain and muscle strength in patients with myofascial pain syndrome: A placebo-controlled trial. J. Phys. Sci..

[25] Karabay İ., Doğan A., Ekiz T., Köseoğlu B.F., Ersöz M. (2016). Training postural control and sitting in children with cerebral palsy: Kinesio taping vs. neuromuscular electrical stimulation. Complement. Clin. Pract..

[26] Voos M.C., Oliveira Tde P., Piemonte M.E., Barbosa E.R. (2014). Case report: Physical therapy managment of axial dystonia. Physioter. Theory Pract..

[27] Park Y.H., Lee J.H. (2016). Effects of proprioceptive sense-based kinesio taping on walking imbalance. J. Phys. Ther. Sci..

[28] Choi Y.K., Nam C.W., Lee J.H., Park Y.H. (2013). The effect of taping prior to PNF treatment on lower etremity proprioception of hemiplegic pateints. J. Phys. Ther. Sci..

[29] Capecci M., Serpicelli C., Fiorentini L., Censi G., Ferretti M., Orni C., Renzi R., Provinciali L., Ceravolo M.G. (2014). Postural rehabilitation and kinesio taping for axial postural disorders in Parkinsons’s disease. Arch. Phys. Med. Rehabil..

[30] Lambert F.M., Malinvaud D., Glaunès J., Bergot C., Straka H., Vidal P.P. (2009). Vestibular asymmetry as the cause of idiopathic scoliosis: A possible answer from Xenopus. J. Neurosci..

[31] Lee J.H., Yoo W.C. (2012). Application of posterior pelvic tilt taping for the treatment of chronic low back pain with sacroiliac joint dysfunction and increased horizontal angle. Phys. Ther. Sport.

[32] Sneag D.B., Bendo J.A. (2007). Pregnancy-related low back pain. Orthopedics.

[33] Kaplan S., Alpayci M., Karaman E., Çetin O., Özkan Y., İlter S., Şah V., Şahin H.G. (2016). Short-term effects of kinesio taping in women with pregnancy-related low back pain: A randomized controlled clinical trial. Med. Sci. Monit..

[34] Kalinowski P., Krawulska A. (2017). Kinesio taping vs. placebo taping in reducing pregnancy-related low back pain: A cross over study. Med. Sci. Monit..

[35] Kamali F., Sinaei E., Taherkhani E. (2018). Comparing spinal manipulation with and without kinesio taping in the treatment of chronic low back pain. J. Bodyw. Ovement Ther..

[36] González-Iglesias J., Fernández-de-Las-Peñas C., Cleland J.A., Huijbregts P., Del Rosario Gutiérrez-Vega M. (2009). Short-term effect of cervical kinesio taping on pain and cervical range of motion in patients with acute whiplash injury: A randomized clinical trial. J. Orthop. Sports Phys. Ther..

[37] Preece H., White P. (2017). Does kinesiology tape increase trunk forward flexion?. J. Bodywork Mov. Ther..

[38] Airaksinen O., Brox J.I., Cedraschi C., Hildebrandt J., Klaber-Moffett J., Kovacs F., Mannion A.F., Reis S., Staal J.B., Ursin H. (2006). European guidelines for the managment of chronic on-specific low back pain. Eur. Spine J..

[39] Álvarez-Álvarez S., José F.G., Rodríguez-Fernández A.L., Güeita-Rodríguez J., Waller B.J. (2014). Effects of kinesio tape in low back muscle fatigue: Randomized, controlled doubled-blinded clinical trial on healthy subjects. J. Back Muscoskelet. Rehabil..

[40] Nelson N.L. (2016). Kinesio taping for chronic low back pain: A systematic review. J. Bodyw. Mov. Ther..

[41] Castro-Sanchez A.M., Lara-Palomo I.C., Matarán-Peñarrocha G.A., Fernández-Sánchez M., Sánchez-Labraca N., Arroyo-Morales M. (2012). Kinesio taping reduces disability and pain slighly in chronic non-specific low-back pain: A randomised trial. J. Phys..

[42] Velasco-Rodan O., Riquelme I., Ferragut-Garcías A., Heredia-Rizo A.M., Rodríguez-Blanco C., Oliva-Pascual-Vaca Á. (2018). Effects of Kinesio Taping Tightness in Low Back Pain. PMR J..

[43] Parreira S.P.C., Costa Lda C., Takahashi R., Hespanhol Junior L.C., Luz Junior M.A., Silva T.M., Costa L.O. (2014). Kinesio taping to generate skin convolutions is not better than sham taping for people with chronic non-specific low back pain: A randomised trial. J. Phys..

[44] Copurgensli C., Gur G., Tunay V.B. (2017). A comparison of the effects of mulligan’s mobilization and kinesio taping on pain, range of motion, muscle strength and neck disability in patient with cervical spondylolysis: A randomized controlled study. J. Back Muscoskelet. Rehabil..

[45] Miller J., Westrick R., Diebal A., Marks C., Gerber J.P. (2013). Immediate effects of lumbopelvic manipulation and lateral gluteal taping on unilateral patellofemoral pain syndrome: A pilot study. Sport Phys. Ther..

[46] Saavedra-Hernández M., Castro-Sánchez A.M., Arroyo-Morales M., Cleland J.A., Lara-Palomo I.C., Fernández-de-Las-Peñas C. (2012). Short-term effects of kinesio taping versus cervical thrust manipulation in patients with mechanical neck pain: A randomized clinical trial. J. Orthop. Sports Phys. Ther..

[47] Rigo M., Weiss H. (2008). The Chêneau concept of bracing—Biomechanical aspects. Stud. Health Technol. Inform..

[48] Paolucci T., Morone G., Di Cesare A., Grasso M.R., Fusco A., Paolucci S., Saraceni V.M., Iosa M. (2013). Effect of chêneau brace on postural balance in adolescent idiopathic scoliosis: A pilot study. Eur. J. Phys. Rehabil. Med..

[49] Misterska E., Glowacki M., Harasymczuk J. (2011). Brace and deformity-related stress level in females with adolescent ideopathic scoliosis based on the bad sobernheim stress questionnaire. Med. Sci. Monit..

[50] Gur G., Dilek B., Ayhan C., Simsek E., Aras O., Aksoy S., Yakut Y. (2015). Effect of a spinal brace on postural control in different sensory conditions in adolescent idiopathic scoliosis: A preliminary analysis. Gait Posture.

[51] Eshraghi A., Maroufi N., Sanjari M.A., Saeedi H., Keyhani M.R., Gholizadeh H., Osman N.A. (2013). Effect of Milwaukee brace on static and dynamic balance of female hyperkyphotic adolescents. Prosthet. Orthot. Int..

[52] Sadeghi H., Allard P., Barbier F., Gatto L., Chavet P., Rivard C.H., Hinse S., Simoneau M. (2008). Bracing has no effect on standing balance in females with adolescent idiopathic scoliosis. Med. Sci. Monit..

[53] Bayar B., Uygur F., Bayar K., Bek N., Yakut Y. (2004). The short-term effects of an exercise programme as an adjunct to an orthosis in neuromuscular scoliosis. Prosthet. Orthot. Int..

[54] Poon K.Y., Li S.M., Roper M.G., Wong M.K., Wong O., Cheung R.T. (2015). Kinesiology type does not facilitate muscle performance: A deceptive controlled trial. Man. Ther..

[55] Cai C., Au I.P., An W., Cheung R.T.I. (2016). Facilitatory and inhibitory effects of Kinesio tape: Fact or fad?. J. Sci. Med. Sport.

[56] Lins C.A., Neto F.L., Amorim A.B., Macedo Lde B., Brasileiro J.S. (2013). Kinesio taping does not alter neuromuscular performance of femoral quadriceps or lower limb function in healthy subjects: Randomized, blind, controlled, clinical trial. Man. Ther..

[57] Bienfait M. (1999). Estudo e Tratamento do Esqueleto Fibroso-Fascias e Pompages.

[58] Cimino S.R., Beaudette S.M., Brown S.H.M. (2018). Kinesio tape influences the mechanical behaviour of the skin of the low back: A possible pathways for functionally relevant effects. J. Biomech..

[59] Lemos T.V., Albino A.C., Matheus J.P., Barbosa Ade M. (2014). The Effect of Kinesio Taping in Forward Bending of the Lumbar Spine. J. Phys. Ther. Sci..

[60] Chang H.Y., Chou K.Y., Lin J.J., Lin C.F., Wang C.H. (2010). Immediate effect of forearm Kinesio taping on maximal grip strength and force sense in healthy collegiate athletes. Phys. Ther. Sport.

[61] Bischoff L., Babisch C., Babisch J., Layher F., Sander K., Matziolis G., Pietsch S., Röhner E., Bischoff L.B. (2018). Effects on proprioception by kinesio taping of the knee after anterior cruciate ligament rupture. Eur. J. Orthop. Surg. Traumatol..

[62] Ridding M.C., Brouwer B., Miles T.S., Pitcher J.B., Thompson P.D. (2000). Changes in muscle response to stimulation of the motor cortex induced by peripheral nerve stimulation in human subjects. Exp. Brain Res..

[63] Maratou E., Theophilidis G. (2000). An axon pacemaker: Diversity in the mechanism of generation and conduction of action potentials in snail neurons. Neuroscience.

[64] De Ru E. (2014). The possibilities of using elastic therapeutic (Kinesio) tape in patients with scoliosis. Scoliosis.

[65] Romano M., Negrini A., Parzini S., Tavernaro M., Zaina F., Donzelli S., Negrini S. (2015). SEAS (Scientific Exercises Approach to Scoliosis): A modern and effective evidence based approach to physiotherapic specific scoliosis exercises. Scoliosis.

[66] Paoloni M., Bernetti A., Fratocchi G., Mangone M., Parrinello L., Cooper M.D.P., Sesto L., Di Sante L., Santilli V. (2011). Kinesio Taping applied to lumbar muscles influences clinical and electromyographic characteristics in chronic low back pain patients. Eur. J. Phys. Rehabil. Med..

[67] Mostafavifar M., Wertz J., Borchers J. (2012). A systematic review of the effectiveness of kinesio taping for musculoskeletal injury. Phys. Sportsmed..

[68] Huang C., Hsieh T., Lu S., Su F. (2011). Effect of kinesio tape to muscle activity and vertical jump performance in healthy active people. Biomed. Eng. Online.

[69] Zhang S., Fu W., Pan J., Wang L., Xia R., Liu Y. (2016). Acute effects of Kinesio taping on muscle strength and fatigue in the forearm of tennis players. J. Sci. Med. Sport.

[70] Choi I.R., Lee J.H. (2018). Effect of kinesiology tape application direction on quadriceps strength. Medicine.

[71] Macedo L.B., Richards J., Borges D.T., Melo S.A., Brasileiro J.S. (2018). Kinesio Taping reduces pain and improves disability in low back pain patients: A randomized controlled trial. Physiotherapy.

